# Yellow Fever Virus Vaccine–associated Deaths in Young Women^1^

**DOI:** 10.3201/eid1710.101789

**Published:** 2011-10

**Authors:** Stephen J. Seligman

**Affiliations:** Author affiliation: New York Medical College, Valhalla, New York, USA

**Keywords:** yellow fever, vaccine, adverse drug reaction reporting systems, women, viruses, dispatch

## Abstract

Yellow fever vaccine–associated viscerotropic disease is a rare sequela of live-attenuated virus vaccine. Elderly persons and persons who have had thymectomies have increased susceptibility. A review of published and other data suggested a higher than expected number of deaths from yellow fever vaccine–associated viscerotropic disease among women 19–34 years of age without known immunodeficiency.

Yellow fever virus (YFV) vaccine had been considered the safest of the live-virus vaccines. Rare neurologic adverse events, called yellow fever vaccine–associated neurotropic disease (YEL-AND), have long been recognized but are seldom fatal. However, in 2001, the vaccine was found to cause a serious, frequently fatal, multisystemic illness, called yellow fever vaccine–associated viscerotropic disease (YEL-AVD), which resembles the illness it was designed to prevent (*1**–**3*). According to reports from the Vaccine Adverse Event Reporting System (VAERS) (www.vaers.hhs.gov), the frequency of YEL-AVD in US vaccinees was 0.4 per 100,000 doses of vaccine administered (*4*).

Elderly persons (*4*) and patients who have undergone thymectomies secondary to thymoma (*5*) are recognized as groups at risk for YEL-AVD. However, several case reports of YEL-AVD in young women raise concern that women of childbearing age might also be at increased risk (*6**–**10*).

## The Study

To investigate the possibility of age- and sex-specific risk groups, a comprehensive YEL-AVD dataset (Table 36-30 in *11*), was analyzed (Figure). This dataset has the advantage of having been compiled with information that is not otherwise publicly available: data from the Centers for Disease Control and Prevention (Atlanta, GA, USA), patient charts, and vaccine manufacturers (T.P. Monath, pers. comm.).

**Figure:**
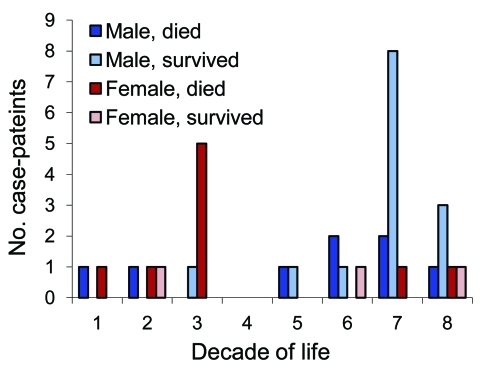
Cases of yellow fever vaccine–associated viscerotropic disease, by patient age, sex, and outcome. One woman who died and whose precise age is unknown was a young woman (P. Vasconcelos, pers. comm.) arbitrarily depicted as being 23 years of age. Data obtained from Table 36-30 in (*11*).

Two concentrations of cases were evident: cases in men >60 years of age who survived and in women 19–34 years of age who died. Although selection bias may have influenced the cases reported, the sex-specific survival rates for these 2 age groups statistically differed: 21% (3/14) versus 0% (0/6) (p = 0.002 by Fisher exact test). In addition to the surprisingly low case-fatality rate for elderly men, only 2 of the 4 patients who had undergone thymectomy and had YEL-AVD died.

Searches for additional YEL-AVD cases among women of childbearing age (15–44 years) and of comparably aged men included review of published cases through PubMed (www.ncbi.nlm.nih.gov/sites/entrez) and reports from the ProMED Web site (http://apex.oracle.com/pls/otn/f?p=2400:1000:). In follow-up of a ProMED listing, 1 case was supplied by Bio-Manguinhos (Rio de Janeiro, Brazil), a producer of YFV vaccine. VAERS also was searched. Information was sought from authors of case reports. Cases listed in VAERS were excluded if another explanation for the adverse event was evident in the case description or if they contained insufficient information to classify the event as YEL-AVD.

A total of 9 fatal cases of YEL-AVD in young adults, all women, were found (Table). Six cases were included in the report by Monath et al. (*11*), and 3 cases were found through the author’s search. The eldest of the 9 case-patients was 34 years of age. One case listed by Monath et al. occurred in 1975 and was originally thought to be yellow fever but was documented as vaccine-related ≈2 decades later (*13*). This patient’s age is not known, but she was reported to be a young woman (P. Vasconcelos, pers. comm.).

**Table:** Characteristics of fatal yellow fever vaccine–associated viscerotropic disease in women of childbearing age who had no known immunologic defects*

Age, y	Country	Year	Vaccine	Days after vaccination		Possible predisposing factors	Virus detection	Neutralizing antibody test results	Reference
				Onset	Death				
19	Brazil	2001	17DD†	2	10	None known	+ RT-PCR liver and spleen		(*12*)
22	United States	2002	17D-204 YF-Vax‡	2	10	None known	YF viral antigen in multiple organs, i.e., liver, lungs, brain, heart, spleen, kidney, lymph nodes		(*7*)
22	United States	2005	17D-204 YF-Vax‡	2	11	See text for postmortem description of thymus	Plasma virus 1.1 × 10^5^ PFU/mL	2,560, day 10	(*9*)
22	Brazil	2000	17DD†	4	11	Hepatitis A and nephritis as a child	+ Culture	IgM +	(*2*)
23	Peru	2007	17DD†	1	9	Acne rosacea	Viral RNA lung 7.6 × 10^6^ and serum 3.9 × 10^6^ PFU equivalents/mL	160 (by PRNT), day 9	(*10*)
Young adult §	Brazil	1975	17DD†	5	9	None known	+ Culture		Table 36-30 in (*11*), (*13*)
24	Peru	2007	17DD†	<1	14	Egg allergy	Viral RNA liver 1.1 × 10^4^ and brain 4.2 × 10^3^ PFU equivalents/mL	10,240 (by PRNT), day 11	(*10*)
26	Spain	2004	17D-204¶	4	10	None known	+ Culture liver, kidney, plasma; real-time PCR liver 6.2 × 10^9^ genome equivalents/g	512 (by microneutralization assay), day 8	(*8*)
34	Brazil	2009	17DD†	1	11	None known	RT-PCR + d 10		#

*+, positive test result; RT-PCR, reverse transcription PCR; YF, yellow fever; Ig, immunoglobulin; PRNT, plaque-reduction neutralization test. Blank cells indicate information is not in the reference cited. †Bio-Manguinhos, Rio de Janeiro, Brazil. ‡Sanofi Pasteur, Swiftwater, PA, USA. §The age of this patient is not known, but she was a young woman (P. Vasconcelos, pers. comm.). ¶Stamaril, Sanofi Pasteur, Lyon, France. #R. Menezes-Martins, pers. comm.

Three fatal cases of possible YEL-AVD among young women reported in VAERS were excluded from the Table because information was insufficient to document the diagnosis. Two other cases of suspected YEL-AVD, 1 each in an 18-year-old man and a 24-year-old woman, occurred outside the United States. Hence, these patients could have come from regions where yellow fever was endemic and thus might have had wild-type yellow fever.

Also excluded from the Table are 2 cases reported in the published literature: 1 in a 23-year-old woman with a partial C4 deficiency and discoid lupus erythematosis hospitalized with severe YEL-AND and YEL-AVD who survived (*14*) and 1 in a 43-year-old woman with systemic lupus erythematosus who died (*10*). The first was excluded because she survived, had clinical features that included YEL-AND, and had known immunodeficiency. For the second patient, a history of disseminated lupus erythematosis, the relatively long interval (30 days) until death (in contrast to the 9–14 days in the other women), and her older age suggest that her susceptibility to the vaccine differed from those listed in the Table.

In several investigations of YEL-AVD cases, extensive sequence analyses did not indicate any substantial evidence of reversion of the vaccine to virulence (*1**,**10*). Two varieties of YFV vaccine are available: the 17DD vaccine produced in Brazil and used in South America and the 17D-204 vaccine (YF-Vax, Sanofi Pasteur, Swiftwater, PA, USA; and Stamaril, Sanofi Pasteur, Lyon, France) used elsewhere. Six cases listed in the Table occurred in 17DD vaccine recipients in South America, and 3 occurred in persons who received 17D-204 as prospective travelers.

The limited racial information available indicates that cases were not confined to persons of any particular racial group. Of 3 case-patients for whom racial information was available, 1 each was described as Caucasian (*8*), black (*2*), and of Pacific Islander ancestry (*7*).

Despite the known association of thymectomy with YEL-AVD, the only observation on possible thymic disease in the reports of the 9 cases is the statement that, at autopsy of a 22-year-old woman from the United States, the thymus was replaced by fat (Table 36-30 in *11*). However, the accuracy of the finding should be considered in the context that, at surgery, experienced cardiothoracic surgeons may have difficulty in distinguishing thymus from adipose tissue (R.L. Berger, pers. comm.) and that the thymus was not examined histologically (R.V. Ridenour, III, pers. comm.). Because the thymus may be difficult to separate from surrounding adipose tissue and is infrequently a source of disease, pathologists, at least in the United States, do not routinely examine it histologically at autopsy (I. Argani, pers. comm.). Thymic deficiencies such as Sutton thymic dysplasia (fatal viral infection in young women with a dysplastic thymus) (*15*) have yet to be excluded.

## Conclusions

Although accurate denominators are not available for calculating age- and sex-specific incidence of YEL-AVD, the number of fatal YEL-AVD cases among women of childbearing age appears to be higher than expected. Further investigation should include ascertainment of family history; exploration of contraceptive medications or occult pregnancy as possible predisposing factors; examination of the thymus at postmortem, including thymus weight and histology; further evaluation of possible complement defects; and evaluation of any associations with autoimmune disease.
